# EFMSDTI: Drug-target interaction prediction based on an efficient fusion of multi-source data

**DOI:** 10.3389/fphar.2022.1009996

**Published:** 2022-09-23

**Authors:** Yuanyuan Zhang, Mengjie Wu, Shudong Wang, Wei Chen

**Affiliations:** ^1^ School of Information and Control Engineering, Qingdao University of Technology, Qingdao, Shandong, China; ^2^ College of Computer science and Technology, China University of Petroleum (East China), Qingdao, Shandong, China

**Keywords:** drug-target prediction, multi-source data, topology and semantic graph, similarity network fusion, selective and weighted fusion

## Abstract

Accurate identification of Drug Target Interactions (DTIs) is of great significance for understanding the mechanism of drug treatment and discovering new drugs for disease treatment. Currently, computational methods of DTIs prediction that combine drug and target multi-source data can effectively reduce the cost and time of drug development. However, in multi-source data processing, the contribution of different source data to DTIs is often not considered. Therefore, how to make full use of the contribution of different source data to predict DTIs for efficient fusion is the key to improving the prediction accuracy of DTIs. In this paper, considering the contribution of different source data to DTIs prediction, a DTIs prediction approach based on an effective fusion of drug and target multi-source data is proposed, named EFMSDTI. EFMSDTI first builds 15 similarity networks based on multi-source information networks classified as topological and semantic graphs of drugs and targets according to their biological characteristics. Then, the multi-networks are fused by selective and entropy weighting based on similarity network fusion (SNF) according to their contribution to DTIs prediction. The deep neural networks model learns the embedding of low-dimensional vectors of drugs and targets. Finally, the LightGBM algorithm based on Gradient Boosting Decision Tree (GBDT) is used to complete DTIs prediction. Experimental results show that EFMSDTI has better performance (AUROC and AUPR are 0.982) than several state-of-the-art algorithms. Also, it has a good effect on analyzing the top 1000 prediction results, while 990 of the first 1000DTIs were confirmed. Code and data are available at https://github.com/meng-jie/EFMSDTI.

## 1 Introduction

The accurate prediction of DTIs is worth that improves the speed and accuracy of new drug discovery. Although the traditional experimental methods have made some progress in DTIs identification, it is a costly, time-consuming process with a high failure rate (Whitebread et al., 2005; Avorn, 2015; Zhao et al., 2018; Liu et al., 2019). With the popularity of artificial intelligence concepts and technologies such as machine learning, more and more researchers are applying machine learning to predict DTIs, dramatically accelerating the new drug development process and revolutionizing the traditional drug development process. It provides accurate drug candidates for drug discovery, further reducing the cost and time of drug discovery. Currently, many researchers have focused on DTI prediction and achieved remarkable achievements through machine learning and deep learning (Li et al., 2016; Ezzat et al., 2019; Abbasi et al., 2020; Bagherian et al., 2021).

Over recent years, a substantial number of computational methods have been developed for predicting drug discovery (Zhu et al., 2005; Sousa et al., 2006; Keiser et al., 2007; Bleakley and Yamanishi, 2009; Buza and Peška, 2017; Luo et al., 2017; Cheng et al., 2018; Olayan et al., 2018; Wan et al., 2018; Yan et al., 2019; Zeng et al., 2019; Wang et al., 2020a; Tang et al., 2020; Zeng et al., 2020; An and Yu, 2021; Chu et al., 2021; Yan et al., 2021; Zong et al., 2021). Target-based (Sousa et al., 2006), ligand similarity-based (Keiser et al., 2007) and machine learning-based (Zhu et al., 2005) methods are the three main-stream in prediction methods. However, obtaining the 3D structure of the protein is very time-consuming, making it difficult to use the target-based approach on a genome-wide scale. Similarly, the ligand-based target prediction usually depends on the structural characteristics of known target ligands. However, the number of known ligands from a single data source for the target protein is insufficient, and the prediction results of ligand-based methods may become unreliable. Currently, with the increasing availability of public data sets, the prediction of DTIs based on machine learning methods has been widely proposed and applied in recent years (Bleakley and Yamanishi, 2009). Moreover, huge amounts of multi-source data are being used to study the properties of drugs and targets to predict DTIs (Tao et al., 2022; Wang et al., 2022).

In the calculation strategy of DTIs prediction, multiple drug and target data sources are often considered. Multi-source data of drugs and targets contain their inherent features and network topology information based on other attributes such as drug side effects. It is found that topology and semantic information often play different roles in the prediction task by analyzing the topological structure and feature graphs using the attention mechanism (Wang et al., 2020b). Tang et al. (2020) provided a marginalized denoising model to predict DTIs by calculating the similarities of target protein sequences and drug chemical structure. Chu et al. (2021) developed DTI-CDF model to predict DTIs through integrating target protein sequences and three drug side effects datasets. It uses multiple data sources to analyze drugs and targets. However, only the side effect and target protein sequences of drugs were considered, and other information about drugs and targets, such as the molecular structure of drugs and disease target correlation, was not considered, which may lose part of the information of drugs targets, resulting in inaccurate DTIs prediction. Olayan et al. (2018) proposed DDR model based on multi-source data of drug and target. It contains eight drug similarity networks and eight target similarity networks, which consider both topology and semantic in-formation. Although the fusion of multi-source data is considered, the contribution of different data sources is not considered. Zeng et al. (2020) proposed deepDTnet model based on multi-source data to predict DTIs. The feature vectors of drugs and tar-gets were learned and spliced for multi-source data of drugs and targets. They treated data from different sources equally, but different data often play different roles in DTIs prediction. An et al. proposed NEDTP model based on network embedding framework (An and Yu, 2021). They applied a random walk to extract the information of each node in the network and learn it as a low-dimensional vector. Finally, the GBDT model was constructed to complete the classification task. Although they consider how to extract and merge multi-source data, they learned the embedding features of drugs and targets by treating random walking paths through different networks equally, without fully considering the contributions of different data sources. Therefore, effectively fusing multi-source data is a challenge for accurately identifying DTIs through considering the topological and semantic information of multi-source data and exploring the weight of different networks. Yan et al. (2019) proposed MKLC-BiRW model to predict DTIs. Although they integrated multi-source heterogeneous data based on the kernel idea of KronrLS-MKL algorithm, they did not comprehensively organize the related data of drugs and targets.

In this paper, we propose a framework named EFMSDTIs to predict DTIs based on the effective fusion of multi-source data. Specifically, EFMSDTI constructed similarity networks of multi-source drugs and targets from heterogeneous data, including the biological characteristics, molecular structure, biological function of drugs and targets. By classifying the different source data of drugs and targets, the drug and target similarity network is divided into the semantic graphs and topology graphs. We propose a selection and weighted entropy fusion algorithm based on SNF (Wang et al., 2014) for the semantic and topology graphs. Network embedding algorithm extracts low-dimensional features of drugs and targets. Finally, the features of drugs and targets learned were input into the prediction model to improve the prediction accuracy of DTIs. The results show that EFMSDTI has better performance over several state-of-art algorithms by classifying the data and treating the classified data with different weights during fusion.

## 2 Materials and methods

### 2.1 Overview of the EFMSDTI method

**FIGURE 1:**
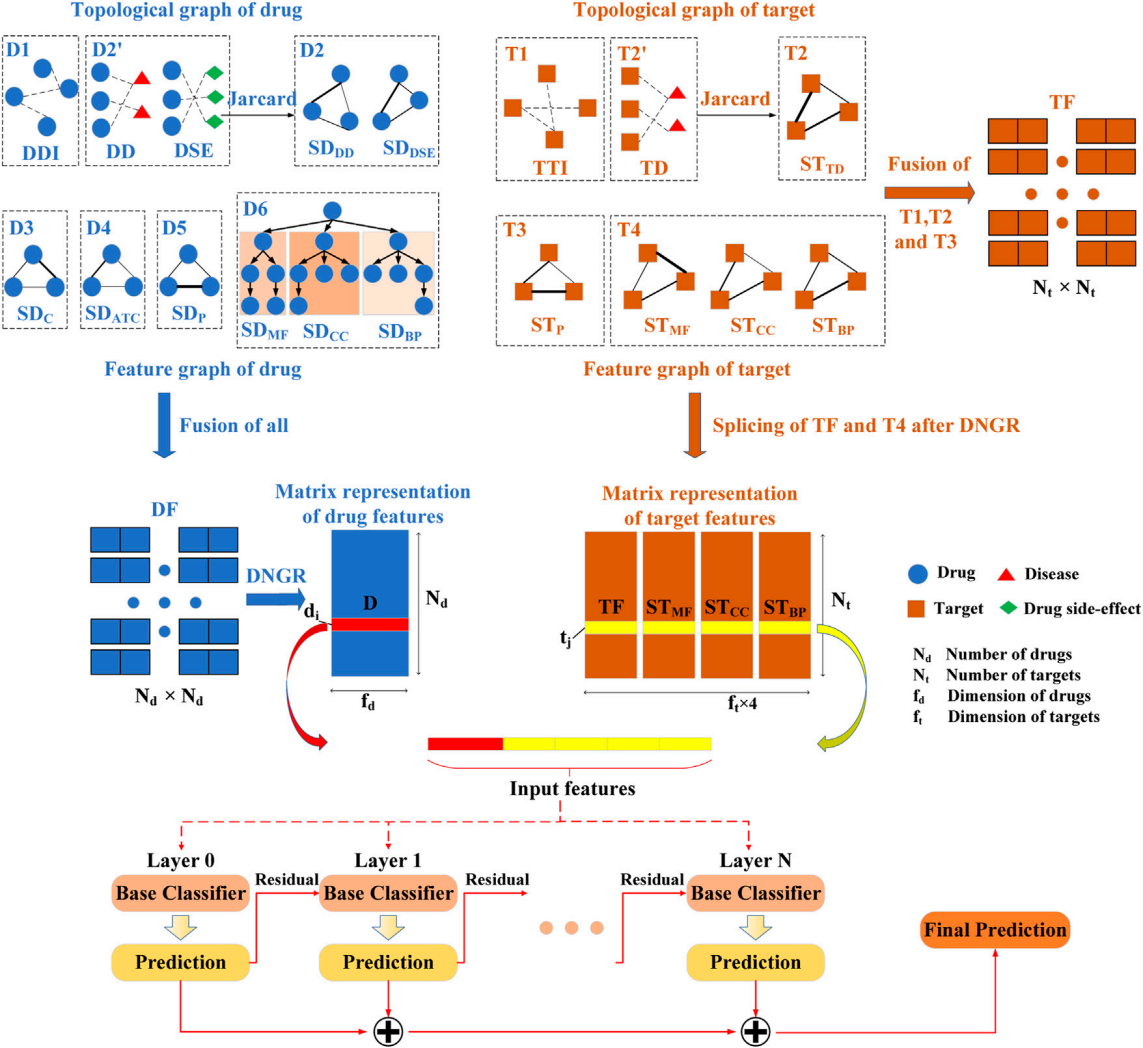
EFMSDTI framework of predicting DTIs. EFMSDTI constructs 15 drug-related networks and target-related networks from heterogeneous multi-source data. Based on the contribution of the class network, the drug and target networks are fused or spliced after the network embedding. Through selective and weighted fusion based on SNF and extract low-dimensional vector of drugs and targets, then features are input into the LightGBM to predict DTIs. Among them, drug-related networks are DDI (Drug-drug), DD (Drug-disease), DSE (Drug-sideEffect), SD_C_ (Chemical similarities), SD_ATC_ (ATC similarities), SD_P_ (Drug targets sequence similarities), SD_MF_ (molecular function similarities), SD_CC_ (cellular component similarities) and SD_BP_ (biological process similarities), target-related networks are TTI (Target-target-interaction), TD (Target-disease), ST_P_ (Target sequence similarities), ST_MF_ (molecular function similarities), ST_CC_ (cellular component similarities) and ST_BP_ (biological process similarities).

Considering the contribution of different source data to DTIs prediction, a framework called EFMSDTI is proposed to predict DTIs. In Figure 1, firstly, multi-source data of drugs and targets can be fused (including selective fusion and weighted fusion) or spliced by classifying multi-source data of them (see Results). For original data, it includes the topological graph (such as Drug-drug, Drug-disease, Drug-side Effect, Target-target, and Target-disease) and semantic graph (such as Drug similarities and Target similarities). According to the biological characteristics of the drug or target, drug or target related networks are divided into several categories, respectively. When there are multiple networks in a category, whether to fuse them into one network is determined according to the contribution of them to DTI prediction. Secondly, the networks are embedded to obtain the low-dimensional representations of drugs and targets based on the Deep neural Networks model for Graph Representations (DNGR) (Shaosheng et al., 2016), respectively. Finally, LightGBM (Qi, 2017) is used to predict the potential DTIs. EFMSDTI has the advantage that a selective weighted fusion algorithm based on similarity fusion is proposed according to the contribution of different source data to DTIs prediction. The aim is to explore an optimal scheme for predicting DTIs by classifying drugs and targets from multiple data sources according to their topology and semantic graphs.

### 2.2 Data source

The drug and target are collected from the DrugBank database (v4.3) (Wishart et al., 2017), the Therapeutic Target Database (Yang et al., 2016), and the PharmGKB database (Hernandez-Boussard et al., 2008). Specifically, bioactivity data for drug–target pairs are collected from ChEMBL (v20) (Gaulton et al., 2012), BindingDB (Liu et al., 2007), and IUPHAR/BPS Guide to PHARMACOLOGY (Pawson et al., 2014). A total of 4978 DTIs are used to build a drug-target interaction network by 732 FDA-approved drugs and 1915 unique human targets (proteins), which include nine different data describing drugs and six data describing targets which refer to Zeng’s work (Zeng et al., 2020). The goal is to analyze the properties of drugs and targets from as many aspects and perspectives as possible in order to improve the prediction accuracy of DTIs.

#### 2.2.1 Drug-related networks

For drugs, there are nine drug-related networks: (i) drug-drug interaction (DDI), (ii) drug-disease (DD), (iii) drug side-effect (DSE), (iv) chemical similarity (SD_C_), (v) Anatomical Therapeutic Chemical (ATC) (Cheng et al., 2013) similarity (SD_ATC_), (vi) protein sequence similarity (SD_P_), (vii) Go (SD_GO_) molecular function similarity (SD_MF_), (viii) Go cellular component similarity (ST_CC_), (ix) Go biological process similarity (ST_BP_). Where (ii) and (iii) calculate their similarity through the Jaccard coefficient.

#### 2.2.2 Target-related networks

For targets, there are six target-related networks: (i) target-target interaction (TTI), (ii) target-disease (TD), (iii) target protein sequence similarity (ST_P_), (iv) Go (ST_GO_) molecular function similarity (ST_MF_), (v) Go cellular component similarity (ST_CC_), (vi) Go biological process similarity (ST_BP_). Where (ii) calculates its similarity through the Jaccard coefficient.

#### 2.2.3 Drug-target interaction network

The drug-target interaction network is described by a bipartite graph G={D,T,R} , where $D=\left\{d_{1},d_{2},\cdots ,d_{n}\right\}$ , $T=\left\{t_{1},t_{2},\cdots ,t_{m}\right\}$ and $R=\left\{r_{ij}|i\in d_{i},j\in t_{j}\right\}$ describes the interaction between drug $d_{i}$ and target $t_{j}$ , and $r_{ij}=\left\{0,1\right\}$ , where $r_{ij}=1$ indicate that drug $d_{i}$ interacts with target $t_{j}$ .

### 2.3 Biometric classification of networks from multiple information

In order to facilitate and effectively evaluate the contribution of different source data to DTI prediction, we divide them into topology and semantic graphs according to the information described by the network manually. Take drugs for example, the drug-drug similarity network, which is obtained through the association of various drugs, is considered as a topological graph; and drug-drug similarity networks based on drug attributes, such as drug molecular structure and drug classification, are considered as semantic graphs. Therefore, the association networks, including DDI, DSE, DD, TTI and TD, are the topology graphs of drugs and targets. The similarity networks between drugs (or targets) calculated based on multi-attribute information of drugs (or targets) are considered semantic graphs.

#### 2.3.1 Target classification

The target-related networks contain two types of graphs: one is the topological graphs of the target, including the TTI and the TD, and the other is the semantic graphs of the target, including the ST_P_ and the ST_GO_. The above topology and semantic graphs are further subdivided according to the described topology information and semantic similarity:

• The first category T1 is the TTI, there is a relationship of interaction between targets. If there is an interaction between $t_{i}$ and $t_{j}$ , then $TTI_{ij}=0$ , otherwise, $TTI_{ij}=0$ ;
• The second category T2 is the TD, it describes the relationship between the target and the disease. Where, TD∈{0, 1} , $TD_{ij}=1$ means that the target is associated with the disease;
• The third category T3 is the similarity network ST_P_, it describes the similarity of protein sequences. The ST_P_ is calculated by the Smith-Waterman algorithm (Smith and Waterman, 1981) based on target sequence;
• The fourth category T4 is the similarity networks ST_GO_, it describes the function similarity of targets. It contains three networks: ST_MF_, ST_CC_ and ST_BP_ which are calculated by a graph-based semantic similarity measure algorithm (Wang et al., 2007). The target classification is shown in Table 1.

**TABLE 1:** Classification of target-related data.

	Target category	Networks
Topological graphs	T1	Target-target network TTI
	T2	Target-disease network TD
Semantic graphs	T3	Similarity network of target protein sequence ST_P_
	T4	Three networks based on GO, ST_MF_, ST_CC_ and ST_BP_

#### 2.3.2 Drug classification

For drugs, the topological graphs include DDI, DD and DSE, the semantic graphs are SD_C_, SD_ATC_, SD_P_ and SD_GO_. The network corresponding to the topology and semantic graphs of target are further subdivided into six categories:

• The first category D1 is the DDI. It is mainly determined by clinical data;
• The second category D2 are the DD and DSE. Drug similarity based on disease and side effects describes drug characteristics from the topological perspective of disease and side effects, respectively;
• The third category D3 is the SD_C_. The $SD_{c}\in \left\{0,1\right\}$ is calculated based on the molecular structure of drug pairs;
• The fourth category D4 is the SD_ATC_. The $SD_{ATC}\in \left\{0,\;1\right\}$ is calculated based on Jaccard coefficient, and averaging ATC classification codes of each drug are up to five levels;
• The fifth category D5 is the SD_P_, it describes the sequence similarity of targets associated with different drugs. The protein sequence similarity between drug pairs (SD_P_) is the average of the similarity of all drug targets;
• The sixth category D6 is the SD_GO_. It describes the function similarity of targets associated with different drugs, including the similarity networks SD_MF_, SD_CC_ and SD_BP_. Similar to the SD_P_, the GO similarity SD_GO_ of drug $d_{i}$ and drug $d_{j}$ is calculated by averaging the GO similarity of all drug targets. The drug classification is shown in Table 2.

**TABLE 2:** Classification of drug-related data.

	Drug category	Networks
Topological graphs	D1	Drug-drug network DDI
	D2	Drug-disease network DD and drug-side effect network DSE
Semantic graphs	D3	Drug chemical similarity network SD_C_
	D4	Drug ATC similarity network SD_ATC_
	D5	Drug-associated protein sequence similarity network SD_P_
	D6	Three networks based on GO, SD_MF_, SD_CC_ and SD_BP_

### 2.4 Selection and weighted fusion networks based on similarity network fusion

In order to fuse different categories of multi-source data more effectively, we use fusion and splicing methods respectively to achieve drug and target feature representation according to the contribution of the different networks to DTI prediction. Among them, the fusion method of multiple networks in this paper is based on SNF algorithm. The SNF solves a multi-source problem by constructing networks of samples (e.g., drug) for each data type and then efficiently fusing these into one network. The procedure of the algorithm is shown in Table 3.

**TABLE 3:** The process of six classes of drug networks by using SNF algorithm is described in algorithm 1.

Algorithm 1: SNF_drug
Input:DDI.txt, DD.txt, DSE.txt, SD_C_.txt, SD_P_.txt, SD_ATC_.txt, SD_GO_.txt Output: FuDrug.mat Begin
1. Compute the similarity matrix of heterogeneous association matrix based on $Sim\left(d_{i},d_{j}\right)$ , including DD and DSE: for b 1 to 2 by 1 step do $Sim\left(d_{i},d_{j}\right)=\frac{\left|disease_{d_{i}}\cap disease_{d_{j}}\right|}{\left|disease_{d_{i}}\cup disease_{d_{j}}\right|}$ end for;
2. Calculate edge wights matrix $W_{l}\left(d_{i},d_{j}\right)$ , normalized matrix $E_{l}\left(d_{i},d_{j}\right)$ and local affinity matrix $S_{l}\left(d_{i},d_{j}\right)$ of each network: for l 1 to L by 1 step do Compute $W_{l}\left(d_{i},d_{j}\right)$ based on Eqs 1, 2; Compute $E_{l}\left(d_{i},d_{j}\right)$ based on Eq. 3; Compute $S_{l}\left(d_{i},d_{j}\right)$ based on Eq. 4; end for;
3. Each similarity network is updated t times iteratively based on $E_{l}^{i}$ : for i 1 to t by 1 step do for l 1 to L by 1 step do Compute $E_{l}^{i}$ based on Eq. 5; end for;
4. After t iterations, calculating the population state matrix $E^{\left(G\right)}$ based on Eq. 6. End.

Suppose that there are L drug networks, let $M_{i}$ represents the adjacency matrix of the drug network l={1,2,⋯,L} . The matrix element $W_{l}\left(d_{i},d_{j}\right)$ is defined as follows:

$$W_{l}\left(d_{i},d_{j}\right)=\exp\left(\frac{-\rho^{2}\left(d_{i},d_{j}\right)}{\mu \epsilon_{i,j}}\right),$$ (1)

where $\rho^{2}\left(d_{i,}d_{j}\right)$ represents the Euclidean distance between drug $d_{i}$ and $d_{j}$ , $d_{i}$ is the vector of similarity between the i -th drug and all the other drugs, μ is a hyperparameter and we recommend setting µ in the range [0.3, 0.8]. Note that while this distance measure works for continuous variables, we recommend using chi-square distances for discrete variables and protocol-based measures for binary variables. $\epsilon_{i,j}$ is described as follows.

$$\epsilon_{i,j}=\frac{mean\left(\rho \left(d_{i},N_{i}\right)\right)+mean\left(\rho \left(d_{j},N_{j}\right)\right)+\rho \left(d_{i},d_{j}\right)}{3},$$ (2)

where $N_{i}=\left\{d_{j}|M_{l}\left(d_{i},d_{j}\right)>0\right\}$ represents the neighbors of the drug in the network l , mean $\left(\rho \left(d_{i},N_{i}\right)\right)$ is the average value of the distances between $d_{i}$ and its neighbors.

To compute the fused matrix from multiple types of measurements, we need to project multiple data types into the same space. Thus, all data types are normalized by calculating matrix $E_{i}$ . The normalized matrix $E_{l}$ is described as follows,

$$E_{l}\left(d_{i},d_{j}\right)=\left\{ \begin{array}{l} \frac{W_{l}\left(d_{i},d_{j}\right)}{2\sum_{a\neq i}W_{l}\left(d_{i},d_{a}\right)},j\neq i\\ \frac{1}{2},\;j=i \end{array}\right.,$$ (3)

where the matrix $E_{l}$ is not affected by self-similarity in the diagonal entries and $\sum_{j}E_{l}\left(d_{i},d_{j}\right)=1$ .

Considering that different drugs tend to have different numbers of neighbors, K nearest neighbors (KNN) is used to measure local affinity as:

$$S_{l}\left(d_{i},d_{j}\right)=\left\{ \begin{array}{l} \frac{W_{l}\left(d_{i},d_{j}\right)}{\sum_{k\in N_{i}^{KNN}}W_{l}\left(d_{i},d_{k}\right)},j\in N_{i}^{KNN}\\ 0,otherwise \end{array}\right.,$$ (4)

where $N_{i}^{KNN}$ represents K neighbors of drug $d_{i}$ .

After calculating similarity matrixes and local affinity matrixes of drugs under different source data, we iteratively update similarity matrixes as follows:

$$E_{l}=S_{l}\left(\frac{\sum_{b\neq l}E_{b}}{L-1}\right)\times {\left(S_{l}\right)}^{T},l=1,2,\cdots ,L$$ (5)

Finally, we get state matrix $E^{G}$ by calculating the average of matrix $E_{l}$ after t iterations as follows:

$$E^{\left(G\right)}=\frac{\sum_{l=1}^{L}E_{l}^{t}}{L}$$ (6)

According to the contribution of multi-source data of drugs and targets to DTIs prediction, we will adopt three different strategies for data fusion. Detailed description is as follows.

#### 2.4.1 Selective fusion

Considering the high-noise nature of multi-source data, we measure the contribution of each data source to DTIs prediction. In order to avoid the data with low contribution mixing with high noise in the process of data fusion and affecting the prediction accuracy, the data sources with low contribution are deleted (see Results), and the remaining drug and target data are fused using SNF respectively.

#### 2.4.2 Weighted fusion based on entropy

The drug or target similarity network calculated by each data source often contains different information. Thus, we compute the nodes’ average entropy to determine each network’s information. For any matrix $M_{i}$ , the entropy of node $d_{i}$ is defined as:

$$H_{d_{i}}=-\overset{n}{\underset{j=1}{\sum }}p_{ij}\log\left(p_{ij}\right),$$ (7)

$$P_{ij}=\frac{m_{ij}}{\Sigma_{j}^{k}m_{ij}}$$ (8)

where $m_{ij}$ represents a item in the matrix $M_{l}$ . Finally, we get the average entropy of all rows as:

$$H_{l}=\overset{n}{\underset{i=1}{\sum }}H_{i}$$ (9)

We take entropy as the weight and update $M_{l}$ as follows:

$$HW_{l}=M_{l}\times H_{l}$$ (10)

#### 2.4.3 Selective and weighted fusion

Combine the two strategies above, the data are filtered which is based on the feedback of the classification networks’ combined results in Supplementary Table S1 (see Supplementary Material), then similar networks are updated though weighting entropy value of networks.

### 2.5 Low dimensional vectors for learning node features

In this paper, DNGR model is used to learn node features from multi-source networks. It consists of three parts, including random surfing, calculation of Positive Pointwise Mutual Information (PPMI) matrix and feature reduction by Stacked Denoising Auto Encoder (SDAE).

#### 2.5.1 Random surfing

The random surfing model motivated by the PageRank model used for ranking tasks. The nodes in the network are randomly ordered. For a node, there is a transition matrix Tr that captures transition probability between different nodes in a network, where Tr(i,j) represents the transition probability from j to i . It considers a random surfing model with restart which introduces a predetermined restart probability at the initial node of each iteration, which can be diagonalized as follow:

$$p_{k}=\alpha p_{k-1}Tr+\left(1-\alpha \right)p_{0},$$ (11)

where $p_{0}$ is the initial 1-hot vector, it said that the i -th item is 1 and all other items is 0. The row vector $p_{k}$ , whose j -th item indicates the probability of reaching the j -th node after the k transition. Based on Eq. 11, α is the probability that i -th node will continue the random surfing procedure, and (1−α) is the probability that i -th node will back to the original node that restart this procedure. A probabilistic co-occurrence matrix $p_{CO}$ is produced by the accumulation of random walk values in a network where two vertices exist directly or after several jumps.

#### 2.5.2 Positive pointwise mutual information matrix

In random surfing process, the probabilistic co-occurrence matrix $p_{CO}$ is obtained by summing up the matrices P generated by the random surfing between two nodes in a network, either directly or after several jumps. Then, we calculate PPMI matrix as follows (Bullinaria and Levy, 2007):

$$PPMI\left(i,j\right)=\left(\log\frac{Pco\left(i,j\right)\times \sum_{i}\sum_{j}Pco\left(i,j\right)}{\sum_{i}Pco\left(i,j\right)\times \sum_{j}Pco\left(i,j\right)},0\right)$$ (12)

where $\sum_{i}\sum_{j}P_{CO}\left(i,j\right)$ represents the sum of all the elements of the matrix $P_{CO}$ , $\sum_{i}P_{co}\left(i,j\right)$ and $\sum_{j}P_{co}\left(i,j\right)$ represent the sum of row elements values and column elements values respectively.

#### 2.5.3 Stacked denoising auto encoder

Finally, the PPMI matrix is used as the input feature x of SDAE, we get high quality low-dimensional vector representations for nodes from the PPMI matrix. The idea of SDAE is to stack multiple denoising autoencoder (DAE) together to form a deep framework. The SDAE uses layer-by-layer greed training, unsupervised training is carried out on each auto coding layer separately, to minimize the error between the input and the reconstruction results. For each hidden layer, the DAE randomly sets the value of the input node being 0 to add noise to the input data in the input layer to prevent overfitting. For example, for a network, the input matrix PPMI of SDAE is 732*732 dimensions, and the output feature dimension is set by yourself. In this paper, the feature dimension is set as 100 dimensions for a drug or target. The SDAE model minimizes the regularized problem and tackles reconstruction error (Zeng et al., 2020), defined as follows:

$$\min_{\left\{WE_{y}\right\},\left\{b_{y}\right\}}∥x-\hat{x}{∥}_{F}^{2}+\lambda \underset{y}{\sum }∥WE_{y}{∥}_{F}^{2},$$ (13)

where $WE_{y}$ is weight matrix, and $b_{y}$ is the bias vector of layer y∈{1,2,⋯,Y} . Y is the number of layers. λ is hyperparameter and ‖•‖ denotes the Frobenius norm.

### 2.6 Drug target interactions prediction based on LightGBM

LightGBM which is an efficient implementation of Gradient Boosting Decision Tree (Friedman, 2001) (GBDT) is proposed by Microsoft. GBDT is a decision tree-based algorithm, it contains multiple based classifiers. The based classifier of each layer is based on the residual of the training data of the based classifier of the previous layer. According to the residual of a layer, the gradient is calculated to fit the regression tree. Finally, using the principle of addition model, all the trained based classifier are added and integrated into the final decision. Compared to the traditional GBDT model, LightGBM improves the efficiency of training data and the accuracy of DTIs prediction.

## 3 Results

### 3.1 Performance analysis of drug target prediction based on combined multiple networks

In order to measure the DTIs prediction effects of different drugs and target data sources of different classes, pairwise combinations of different drug data and target data are conducted to calculate the DTIs prediction performance. If a type of network contains more than one, we have two ways to learn the features of nodes in the net-work: 1) Multiple networks are fused using SNF, and low-dimensional representations of nodes of the fused network are learned using DNGR; 2) Low-dimensional representations of nodes of each network are learned using DNGR, and then the features of nodes are spliced for multiple networks.

* T4S means the fourth category T4 including three networks select splicing method. D2F and D6F mean the second and sixth category of drugs, including multiple networks that select fusing method.

**FIGURE 2:**
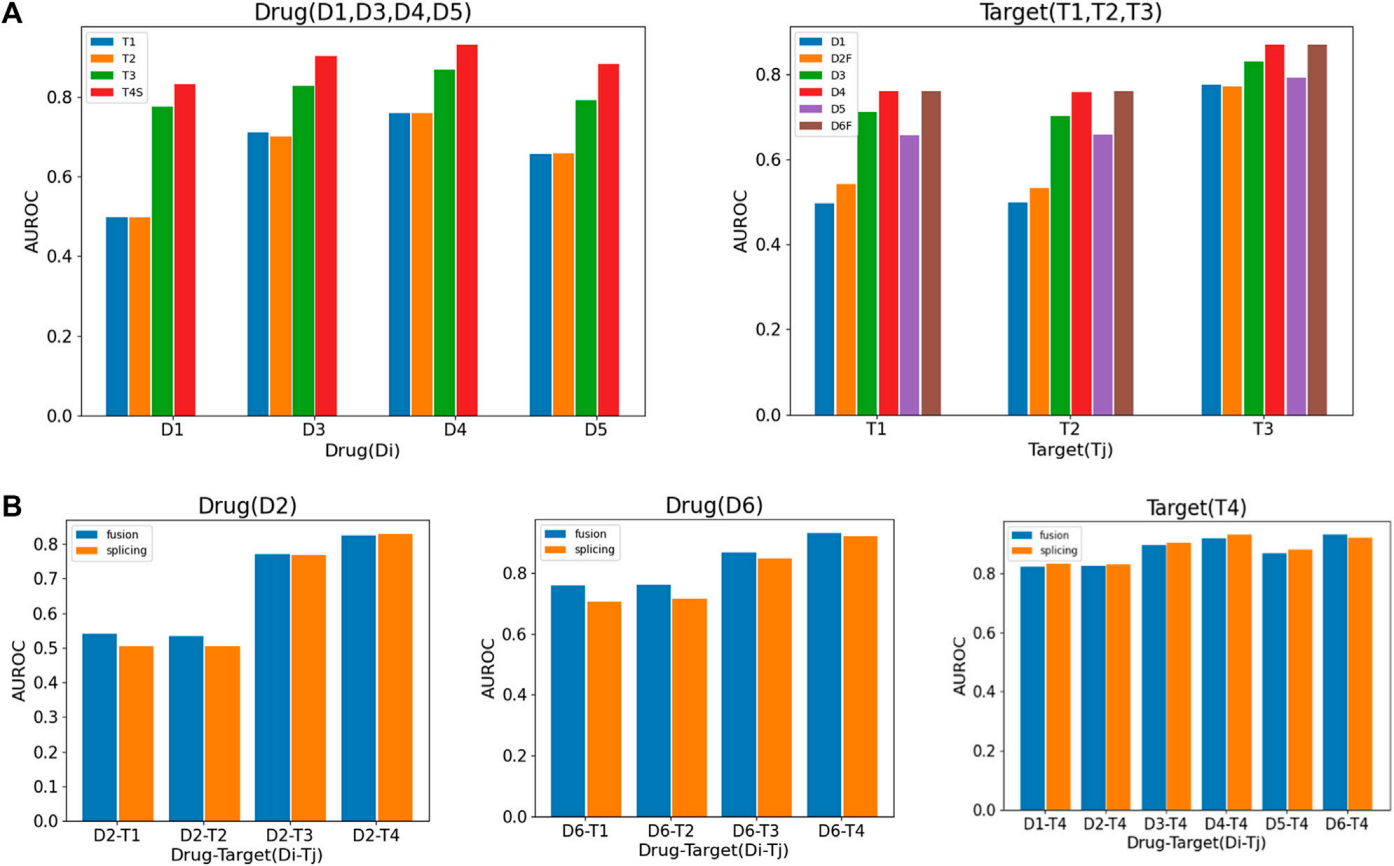
Comparison of DTI prediction accuracy (AUROC) under different drug and target data combinations. It describes the DTIs prediction of six categories of drugs and four categories of targets combinations. **(A)** The DTIs prediction of drugs Di(i=1,3,4,5) which contains one network combine all target class networks and target Tj=(j=1,2,3) which contain one network combine all drug class networks; **(B)** the DTIs prediction of drugs Di=(2,6) and target Tj=(j=4) with fusion and splicing respectively.

Considering that the category networks of drugs D2 , D6 and that of target T4 , we used the above two methods respectively to compare the area under the receiver operating characteristic curve (AUROC) of DTIs combined with different data (Figures 2A,B). It is shown that the contribution of drugs D1 and D2 , targets T1 and T2 to DTIs prediction is much lower than other data. Also, it is found that the fusion method has advantages in DTIs prediction for drugs and that of the splicing method for target (Figures 2A,B). According to the comprehensive analysis of Figure 2, we come to two conclusions about drugs based on the SNF fusion method as follows:

(1) D3 , D4 and D6 of drugs, T3 and T4 of targets have a greater influence on the final results, while D1 , D2 and T1 , T2 which are topological graphs may have unavoidable noise that will affect the accuracy of DTIs prediction.
(2) Fused networks that combine multiple networks in D2 and D6 of drugs have the better performance than that of splicing; while splicing the feature vectors of nodes in each network is slightly better than fusing network in T4 , as shown as in Figure 2B.

Therefore, in the subsequent analysis, fusion and splicing methods are used for D2 , D6 and T4 respectively. Based on the above two conclusions, we consider selective and weighted fusion (see Materials and Methods) to complete the DTIs prediction.

### 3.2 Comparison of drug target interactions prediction performance under different fusion methods

Through the comprehensive analysis of Figure 2, we can see that the AUROC is lower based on D1 , D2 , T1 and T2 in the DTI prediction than others. Although there are better results when D1 , D2 combines with T3 and T4 , and T1 , T2 combines D3 , D4 and D6F , this may be due to the high contribution of T3 , T4 , D3 , D4 and D6F to the final results. Therefore, we think that D1 , D2 , T1 and T2 provide less contribution to DTIs prediction and may introduce high noise.

According to the above results, we complete network fusion with different strategies. For high noise data, different filtering or weighting strategies are used and ablation experiments are performed. Then low-dimensional feature vectors of drugs and targets are learned based on DNGR. Finally, LightGBM was used to obtain a good prediction effect based on selective and weighted strategy, that is, AUROC and AUPR were 0.982.

#### 3.2.1 Selective fusion

To reduce the effect of high noise, we simply filter D1 , D2 , T1 and T2 . Table 4 shows that selective fusion has better effects in two indexes, AUROC and the area under the recall versus precision curve (AUPR) compared to total data fusion. It is shown that reducing the introduction of high noise is indeed helpful in improving the prediction performance. Also, it is found that the optimal combination fusion is obtained when only D1 , D2 of drugs are deleted. It shows that although deleting noisy data can improve performance, simple deletion can also cause some information to be lost.

**TABLE 4:** Prediction performance of selective fusion. For ease of description, abbreviations in the model are expressed as follows.

Model	AUROC	AUPR
DF_TFS	0.903	0.908
DE_D125_DF_TFS	0.918	0.923
DE_D12_T12_DF_TS	0.924	0.933
**DE_D12_DF_TFS**	**0.942**	**0.950**
WEC_DF_TFS	0.903	0.904
**WE_DF_TFS**	**0.904**	**0.905**
**DE_D12_WE_DF_TFP**	**0.982**	**0.982**

*D and T are drug and target; F and S are fusion and splicing; DE represents delete; A D or T followed by numbers indicates what kinds of data is deleted. The abbreviations WE and WEC represent unclassified network-based entropy and classified network entropy respectively.

DE_D12_DF_TFS means that the first class of the drug is deleted, and the remaining drugs are fully fused, and the first, second and third classes of the target are fused to learn the features and then spliced with the fourth class.

WE_DF_TFS means represents the entropy weighting of all the networks of the drug and the target, and all the networks of the drug are fused, and the three types of networks before the target are fused to learn features, and then they are spliced with the fourth type of network.

DE_D12_WE_DF_TFP represents the synthesis of the first two bold methods, that is, the first type of drug network is deleted, all the networks are entropy-weighted, and the drug network is fully fused, and the first three types of target networks are fused to learn features, and then spliced with the fourth type of network.

#### 3.2.2 Weighted entropy fusion

Considering the fact that different data sources may provide different contributions to DTIs prediction, simple deletion may cause information loss, so a weighted fusion of different data sources is carried out. In weighted fusion, we use entropy to evaluate the weighted value of each network during fusion (see Materials and methods). For calculating the entropy of networks, we consider two cases: One is the unclassified networks, and the other is the classified networks. The unclassified networks calculate the entropy of each network by SoftMax, normalized all values of the entropy, and weighted every network before fusing. For the classified network, the difference is that the normalization is based on the classified networks, that is, two or more networks in the classified network contains have to be multiplied by the same weight value. We can see that there is little difference between the two methods of weighted fusion. However, compared with directly deleting the data with low predictive performance, the method based on weighted fusion is significantly lower than the method based on selective fusion.

#### 3.2.3 Selective weighted fusion

Combining the advantages of selection and weighting strategies, a selective and weighted entropy fusion strategy is used. After deleting D1 , D2 and performing weighted fusion on the remaining data, it is found that it has better performance in DTIs than only selective or only weighted fusion (Table 4).

### 3.3 High performance of EFMSDTI

Based on the ablation experimental, the strategy of selective weighted fusion is used to predict DTIs, named EFMSDTI. To evaluate the performance of EFMSDTI, four previous state-of-the-art methods are used for comparison. In a 5-fold cross-validation, 20% of the positive and negative samples are randomly selected as the test set, and 80% of the drug-target pairs are used as the training set. As the Table 5, EFMSDTI, which has the highest AUROC and AUPR, outperforms four previous state-of-the-art methods: NEFTP, NeoDTI, deepDTnet, DTINet. A brief description of these methods is as follows:

**TABLE 5:** Comparison of EFMSDTI with other state-of-the-art methods for DTIs prediction.

Model	AUROC	AUPR
EFMSDTI	**0.982**	**0.982**
NEDTP An and Yu, (2021)	0.971	0.967
deepDTnet Zeng et al. (2020)	0.963	0.969
NeoDTI Wan et al. (2018)	0.97	0.91
DTINet Luo et al. (2017)	0.932	0.943

#### 3.3.1 NEFTP

A heterogeneous network embedding framework for predicting similarity-based drug-target interactions was performed, which builds a similarity network based on 15 heterogeneous information networks, and applies a random walk to extract the information of nodes in the network and learns low-dimensional vectors. Finally, the classifier predicts DTIs (An and Yu, 2021). It learns node features by treating the random walk paths of different networks equally, but the contributions of different networks are different.

#### 3.3.2 NeoDTI

Integration of neighbor information from a heterogeneous network for discovering new drug-target interactions develop a new nonlinear end-to-end learning model (Wan et al., 2018). The model integrates various information from heterogeneous network data and automatically learns representations that preserve drug and target topologies to facilitate DTI prediction.It focuses on the topological information of drugs and targets, and the collection of feature information is not enough, only drug structure similarity and target sequence similarity. But the characteristic information of drug and target is not limited to these two.

#### 3.3.3 deepDTnet

Target identification among known drugs by deep learning from heterogeneous networks (Zeng et al., 2020) was implemented, which collects 15 information networks to learn the feature vectors of each node in each network, and inputs the PU prediction model to predict DTIs after splicing 15 feature vectors. Similarly, the model treats multi-source data equally, but the contribution of each information network is different.

#### 3.3.4 DTINet

A network integration approach for drug-target interaction prediction and computational drug repositioning from heterogeneous information was implemented, which integrates multi-network information and learns node features through compact feature learning algorithm, and finally inputs DTIs in PU learning. Although it integrates multi-network information, it does not consider the in-depth study of multi-network information fusion.

### 3.4 Validation of the top-ranked predictions

**FIGURE 3:**
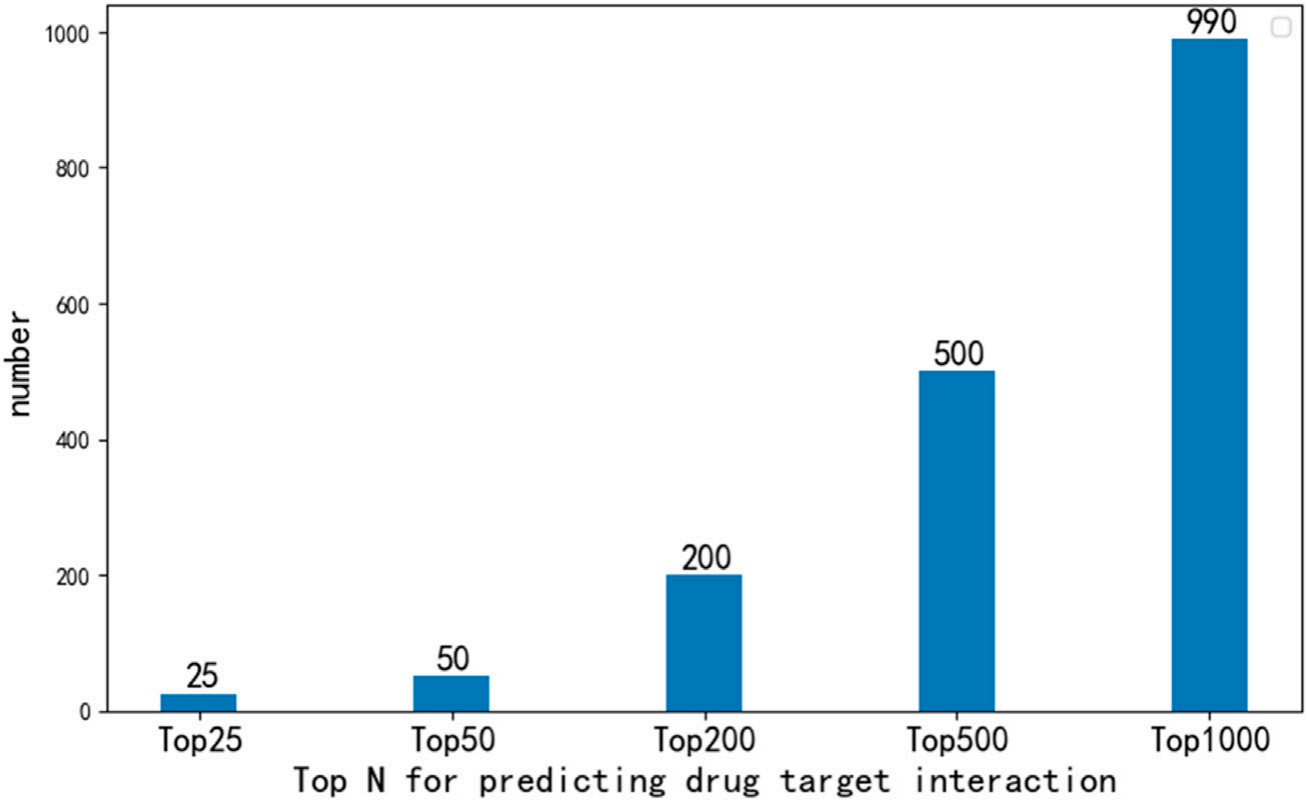
The number of DTIs that were verified to exist in the top 1000 prediction results.

In order to further analyze the performance of EFMSDTI method to identify DTIs, the top-ranked predictions are verified. We select the top 1000 prediction DTIs for each fold, and merge the 5-folds by averaging. The top 1000 prediction DTIs are analyzed in our analysis. As shown in Figure 3, 990 of the top 1000 DTIs are known DTIs. The top 25, 50, 200 and 500 prediction results are known DTIs. Based on the predicted score of the model, the drug-target pairs that do not interact in known DTIs are considered to be novel DTIs according to rank of the predicted value in the top 1000 samples. Ten DTIs in the first 1000 prediction results that are not verified in Table 6.

**TABLE 6:** The 10 unverified DTIs out of the top 1000 prediction results.

DTI’s ranking in the top 1000 predictions	Drug	Target
922nd	DB08882:Linagliptin	2934:GSN
953rd	DB00612:Bisoprolol	3283:HSD3B1
963rd	DB08896:Regorafenib	9453:GGPS1
991st	DB00606:Cyclothiazide	4698:NDUFA5
993rd	DB00602:Ivermectin	834:CASP1
994th	DB01594:Cinolazepam	378:ARF4
995th	DB06204:Tapentadol	55825:PECR
997th	DB00312:Pentobarbital	4326:MMP17
998th	DB01364:Ephedrine	4507:MTAP
999th	DB00459:Acitretin	10606:PAICS

There were 10 sets of drug-target interactions in the first 1000 prediction results that were not verified in Table 6. These DTIs are considered to be potential drug-target interaction. Diseases can be treated with certain drugs, and these drugs are related to the disease. We’re going to test it from different angles. Disease can be caused by the abnormal expression of certain proteins, so this protein is associated with disease. As a result, drugs and targets that share the same disease are thought to be more likely to interact (An and Yu, 2021). The pair of bisoprolol (drug) and HSD3B1 (target) was one of the novel DTIs identified. The HSD3B1 is related to Hypertensive disease and other diseases. Bisoprolol also appears to have effect on this disease: Hypertensive disease. There is also support for drug-target interactions in the database. Ivermectin and caspase-1 (CASP1) was one of the novel DTIs identified. The target prediction module of drug in CHEMBL database was confirmed to be related to caspase-1 (Bosc et al., 2019).

## 4 Discussion

By analyzing and comparing the results of several experiments, we continuously adjust and optimize the prediction model. The results are analyzed and explained, and the optimal model, named EFMSDTI, is obtained based on the currently used data set. The procedure of EFMSDTI is the selective weighted fusing data, extracting the low-dimensional features of drugs and targets, and predicting DTIs using the LightGBM framework. The AUROC value of our final prediction result reached 0.982, which has better performance than several state-of-the-art algorithms.

DTIs prediction requires more accurate analysis of multi-source data of drugs and targets. Multi-source data can improve more comprehensive information than a single data. However, at the same time, multiple data sources may also bring some noise, so the data processing of multiple-source data is essential. Therefore, considering the contribution of different data, an effective fusion method named EFMSDTI is proposed. The result of the comprehensive analysis shows a higher performance of EFMSDTI. Moreover, through the concept of class network, we also found a new angle of the fusion method. In this paper, we use the popular fusion strategies and entropy-based weighted method to improve the prediction accuracy. The multi-source data used in this paper included nine sources for the drugs and six for the targets. According to current studies (Wan et al., 2018; Zeng et al., 2020), data sources for drugs and targets are not limited to this, such as drug-induced gene expression profiles, drug pathways profiles, and so on. In the future, more data sources for drugs and targets will be studied to complement the rich-ness of drugs and targets with multiple networks, and to further confirm our strategy’s robustness. The fused network uses the graph embedding method of DNGR to extract high-quality low-dimensional features in this paper. Currently, there are many other methods to extract features, which may also improve the model’s prediction accuracy.

In this paper, we manually decide the weighted measure according to the test result metric AUROC, which has certain empiricism and is not a perfect weighting for the results. At present, the most popular mechanism is called attention mechanism, which uses machine self-learning to adjust the weighted value of features during the learning process. The mechanism of self-learning by results will also be the content of future research.

## Data Availability

The original contributions presented in the study are included in the article/Supplementary Material, further inquiries can be directed to the corresponding author.
